# *Ctenocephalides canis* is the dominant flea species of dogs in the Republic of Korea

**DOI:** 10.1186/s13071-018-2769-9

**Published:** 2018-03-20

**Authors:** Kyu-Sung Ahn, Shin-Eui Huh, Sang-Woo Seol, Ha-Jung Kim, Kuk-Hyun Suh, SungShik Shin

**Affiliations:** 1 Department of Parasitology, College of Veterinary Medicine, Chonnam National University, 77 Yongbong-ro, Buk-ku, Gwangju, 61186 Republic of Korea; 2 Department of Internal Medicine, College of Veterinary Medicine, Chonnam National University, 77 Yongbong-ro, Buk-ku, Gwangju, 61186 Republic of Korea

**Keywords:** *Ctenocephalides canis*, *Ctenocephalides orientis*, Dog fleas, Epidemiology, CVBD

## Abstract

**Background:**

The status of flea infestation in dogs is an important public health concern because of their cosmopolitan distribution worldwide and the flea-borne disease transmission. In the present study, we investigated the flea infestation among 116 outdoor dogs (57 females and 59 males) in 8 rural areas of Jeonnam Province, Republic of Korea.

**Results:**

Thirty-three dogs (28.4%) were infested with fleas, and all dogs were infested with *Ctenocephalides canis*. One dog from Hampyeong was co-infested with *Ctenocephalides felis orientis*, but no dogs were infested with *Ctenocephalides felis felis*. The reasons behind this almost exclusive distribution of flea species in dogs from Korea are currently unknown and will require further epidemiological and biological investigations. However, since all dogs investigated in the study were raised in an outdoor environment, the development of flea eggs, larvae and pupae in climatic conditions in Korea might have negatively affected the survival of other flea species. Due to the shoes-off culture and floor-heating system of Korean houses, indoor dogs are rarely infested with fleas in Korea.

**Conclusion:**

To our knowledge, this is the first report on the distribution survey of flea species infesting dogs in Korea and the first report of *C. orientis* infesting a dog in Korea.

## Background

The dominant flea species infesting dogs in the USA, Mexico and western Europe is *Ctenocephalides felis felis* while in some countries in central and eastern Europe, as well as Ireland and Argentina the flea species is *Ctenocephalides canis* [1–6]. *Ctenocephalides orientis* (syn. *Ctenocephalides felis orientis*), on the other hand, is prevalent among dogs in Asian countries such as India, Malaysia and Thailand [7–10]. In cats, the major flea species is *C. felis felis* regardless of geographical region [3, 4, 6, 11]. However, unlike in other countries, flea infestation among pet dogs and cats are rarely considered a serious issue among small animal practitioners in Korea and no clinical cases of flea infestation have been reported from client-owned pet dogs or cats. While dogs with allergic dermatitis due to house dust mites are reportedly high in Korea (62/101, 61.4%), positive rate for flea allergens was only 7.9% (8/101) [12]. Koreans customarily take off their shoes when they enter their homes and remain bare-footed indoors. For this reason houses possess an underfloor heating system that contributes to low-humidity conditions that do not favor survival and propagation of fleas [13, 14]. Carpets are rarely covered in the living room. Instead, floors are covered with laminate or hardwood which makes residential houses of Korea, as well as the dogs that live therein, relatively free of fleas.

Unlike flea-free indoor dogs, outdoor dogs do become infested with fleas in Korea. Previously, we investigated the prevalence of ectoparasite infestation among stray dogs in Gwangju City, Republic of Korea from November 2003 to August 2005 and found that 6.8% of 103 dogs in the study was infested with *C. canis* [15].

Flea infestation on dogs is important to control for the prevention of flea-borne diseases in dogs and flea-borne zoonosis to humans. *Ctenocephalides* fleas, especially *C. felis*, are known to transmit a variety of zoonotic pathogens such as *Bartonella henselae*, *Bartonella clarridgeiae*, *Bartonella quintana*, *Rickettsia typhi* and *Rickettsia felis*, which, in humans, can cause cat scratch disease, endocarditis and flea-borne spotted fever, respectively [16–22]. However, an epidemiological survey on flea species in dogs has not been carried out in Korea. In this study, we aim to determine the prevalence and species of flea infesting outdoor dogs in Jeonnam Province in Korea.

## Methods

**Fig. 1:**
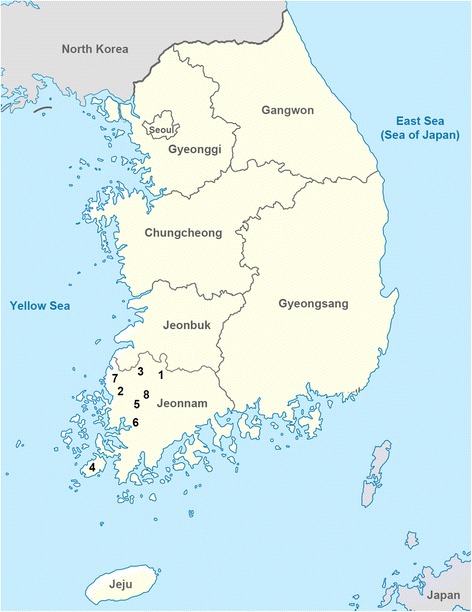
Map of the study area in the southwestern Korean Peninsula. Flea infestation was investigated in dogs from 8 local areas: Damyang (1), Hampyeong (2), Jangseong (3) Jindo (4), Naju (5), Yeongam (6), Yeongwang (7) and SongJeong (8)

Between January 2016 and October 2017, 116 dogs from 8 regions of Jeonnam Province, Korea were examined (Fig. 1). Village households in the study area were randomly visited and if dogs were sighted in the yard, owners were offered a free-health check for their dogs. During this time dogs were inspected for flea infestation using flea comb and visual examination for flea dirt on the skin. Dogs were of random breed, sex and age. All dogs in the study were outdoor dogs that had been kept outdoors in dog kennels and were not raised or allowed to enter their owner’s indoor residential areas. Veterinarians performed an initial inspection of dogs for the presence of fleas on the skin. Dogs were combed over the entire body, but only up to three adult fleas were collected from each dog for identification to minimize the potential bias in the distribution pattern of different flea species due to variations in the flea numbers per dog.

The collected fleas were stored in 70% ethanol and transferred to the Department of Parasitology, Chonnam National University College of Veterinary Medicine. Fleas were mounted on slide glasses in polyvinyl alcohol-lactic acid (PVA) mounting medium [23]. Fleas were sexed and identified to species using a compound microscope with the aid of keys and descriptions from literature [7, 24, 25].

## Results

Out of 116 dogs investigated in this study, 33 dogs (28.4%) were infested with fleas (Table 1). The highest number of flea-infested dogs was recorded in Hampyeong (15/37, 40.5%). At least one dog from each town of Chonnam Province was infested with fleas. A total of 76 adult fleas, 21 males and 55 females, was collected.

**Table 1:** Infestation status of outdoor dogs with fleas in the southwestern regions of Korea

Location	No. of dogs examined			No. of dogs with fleas		No. of fleas collected from dogs											
						*C. canis*			*C. orientis*			*C. felis felis*			Total		
	F	M	Total	Number	Percent	M	F	Total	M	F	Total	M	F	Total	M	F	Total
Damyang	3	4	7	1	14.3	1	2	3	0	0	0	0	0	0	1	2	3
Hampyeong	15	22	37	15	40.5	5	31	36	0	1	1	0	0	0	5	32	37
Jangseong	11	6	17	3	17.6	1	7	8	0	0	0	0	0	0	1	7	8
Jindo	8	5	13	5	38.5	9	2	11	0	0	0	0	0	0	9	2	11
Naju	5	3	8	1	12.5	0	1	1	0	0	0	0	0	0	0	1	1
Yeongam	5	10	15	4	26.7	2	9	11	0	0	0	0	0	0	2	9	11
Yeongwang	5	4	9	1	11.1	0	2	2	0	0	0	0	0	0	0	2	2
SongJeong	5	5	10	3	30.0	3	0	3	0	0	0	0	0	0	3	0	3
Total	57	59	116	33	28.4	21	54	75	0	1	1	0	0	0	21	55	76

*Abbreviations*: *F* female, *M* male

**Fig. 2:**
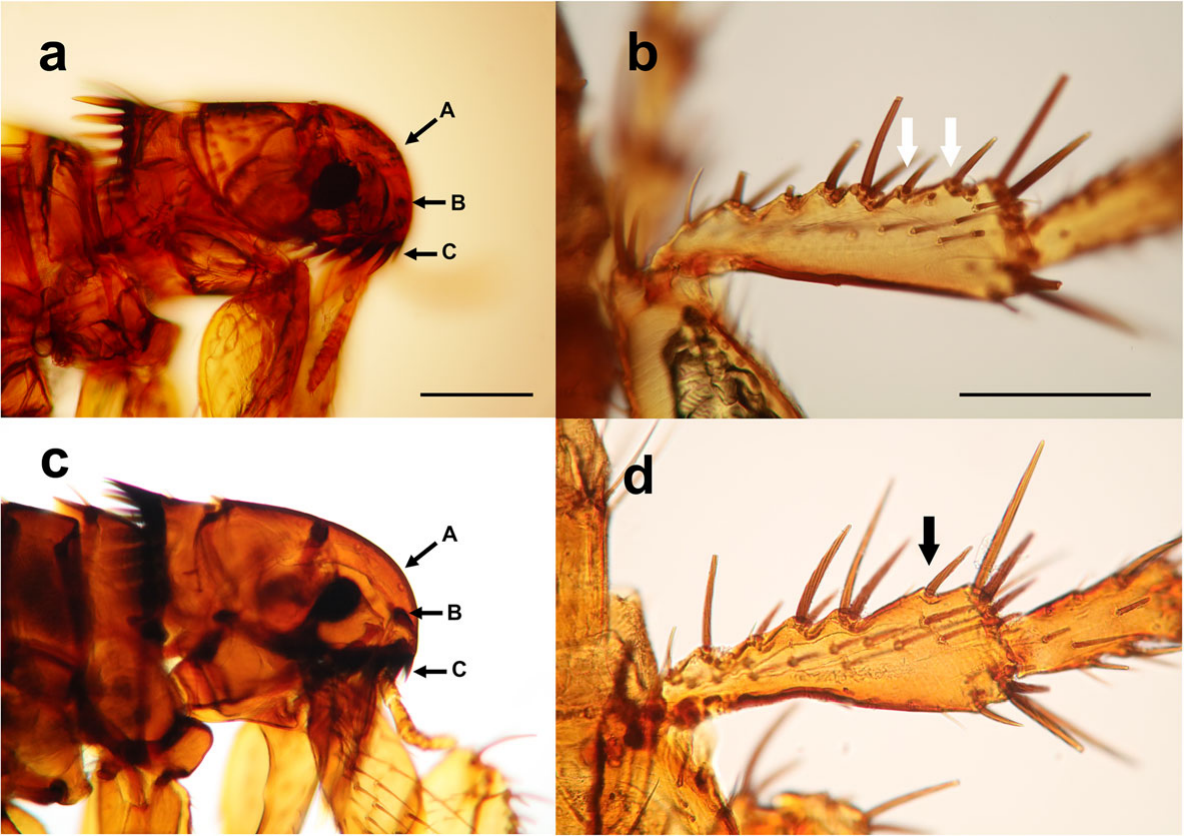
Characteristic features of *Ctenocephalides canis* and *Ctenocephalides orientis* collected from dogs in Korea. **a** *C. canis* with a bluntly rounded frons or head (A) and a short stout dorsal incrassation (B). The first spine (C) of the genal ctenidia was close to half the length of the second spine. **b** The dorso-posterior margin of the hind tibia bore two notches with stout stetae (white arrows) between the post median and apical setae. **c** *C. orientis* was characterized by a round frons (A) and a short, club-shaped dorsal incrassation (B) similar to *C. canis*. The first spine (C) of the genal ctenidia was close to the length of the second spine (not in focus). **d** The dorso-posterior margin of the hind tibia bore one notch with stout stetae (black arrow) between the post median and apical setae

The majority of flea specimens was identified as *Ctenocephalides canis* based on the morphology, which was characterized by a short, sharply vertical frons and a short, club-shaped dorsal incrassation (Fig. 2a). The posterior margin of the hind tibia had two notches bearing stout setae between the post-median and apical setae (Fig. 2b). One dog from Hampyeong was co-infested with *Ctenocephalides orientis*, as characterized by a short, rounded frons and a short dorsal incrassation (Fig. 2c). The dorso-posterior margin of the hind tibia of this species had only one notch bearing a stout seta between the post-median and apical setae (Fig. 2d).

## Discussion

This study clearly shows that a considerably high number of outdoor dogs in Korea is exposed to flea infestation, and almost all flea species from dogs are *C. canis*. The cat flea, *C. felis felis*, is the predominant species found in both cats and dogs worldwide including the USA and Europe [1, 2, 11, 26]. On the other hand, *C. canis* was the most common flea species on dogs from Greece, Ireland and Argentina [3–5]. Since a sample of 116 dogs is not enough to draw conclusions about the exclusive distribution of *C. canis* in Korea and since an exclusive distribution of one species of fleas in a region is rather rare, results of this study call for further epidemiological research before making conclusions on the distribution of flea species in dogs from Korea.

The distribution pattern of *C. felis felis* and *C. canis* worldwide may depend more on the environmental than on-host factors. Most of the flea life-cycle, i.e. eggs, larvae and pupae undergo their developmental cycle off the host on the ground, so the temperature and humidity on the ground and in the dirt may significantly affect the survival of juvenile fleas.

Several previous reports support this assumption in that while *C. felis felis* was generally common among urban and indoor type dogs or cats [5, 27]; studies from Spain, Hungary and Germany indicated that *C. canis* were more common among rural and outdoor animals [1, 28, 29]. Also, wild animals such as red foxes were infested with *C. canis* [30]. The dog flea favors colder climates than the cat flea; thus, this species is more abundant in rural habitats and is largely confined to outdoor dogs [31–33]. According to the accumulative weather data recorded by the national meteorological service of the Republic of Korea (Korea Meteorological Administration, http://www.kma.go.kr/), the annual lowest and highest temperatures of the study area for 30 years between 1988 and 2017 were -9.6 ± 1.8 °C and 35.3 ± 1.4 °C, respectively (average 14.1 ± 0.5 °C). During the winter season, the average temperature for the same period in the study area was 2.4 ± 0.9 °C (lowest -9.6 °C, highest 17.7 °C). The cold winter season of Korea may therefore be too harsh for the juvenile developmental stages of many flea species that are adopted to warm climate, and only those species that can withstand such harsh outdoor weather condition may better be able to survive.

Only one dog in the survey hosted *C. orientis*. Formerly known as *C. felis orientis*, the species appears to be phylogenetically closer to *C. canis* than *C. felis* because *C. canis* formed a sister clade to *C. orientis*, not *C. felis felis*. [24, 34, 35]. *Ctenocephalides orientis* has been reported to be found in Asian countries such as India, Malaysia and Thailand [7–9, 24], and this study is the first report of *C. orientis* found in dogs from Korea. Other subspecies of *C. felis* are geographically restricted, such as *C. felis damarensis* in south-western Africa and *C. felis strongylus* in the Ethiopian zoogeographical region [7].

## Conclusions

We investigated the flea infestation among outdoor dogs raised in eight rural areas of Jeonnam Province, Republic of Korea and showed that 28.4% of dogs were infested with *C. canis*, the dog flea and one dog co-infested with *C. orientis*. No dogs were infested with *C. felis felis*. To our knowledge, this is the first report on the distribution survey of flea species infesting dogs in Korea and the first report of *C. orientis* infesting a dog in Korea.
